# Intracanal adaptation and bond strength assessment of novel liquid/powder calcium silicate-based root canal sealer

**DOI:** 10.4317/jced.64109

**Published:** 2026-08-28

**Authors:** Reem Abdelgawad, Kawthar Bukhari

**Affiliations:** 1 Department of Restorative Dental Sciences, College of Dentistry, Taibah University, Madinah, Kingdom of Saudi Arabia; 2 Department of Endodontics, Faculty of Dentistry, Suez Canal University, Egypt; 3 University Dental Hospital, Taibah University, Madinah, Kingdom of Saudi Arabia; 4 Health and Life Research Center, Taibah University, Madinah, Kingdom of Saudi Arabia

**Keywords:** Bond strength, calcium silicate sealers, root canal treatment scanning electron microscope, sealer adaptation.

## Abstract

**Background:**

Awareness about the vital role the root canal sealer plays in acquiring a three-dimensional seal necessitates continual work to develop a material that encompasses the intended physical, chemical, and biological properties. Therefore, the present study was designed to assess the sealing ability of MTApex powder/liquid and TotalFill ready-to-use calcium silicate-based formulas via measuring their adaptation and bond strength to the root canal wall. The potentiality of root canal sealer to adapt and bond with dentin wall is a suggestive caliber for obturation quality assessment. Calcium silicate-based root canal sealers gained broad acceptance due to their distinctive properties and clinical performance. MTApex is a novel calcium silicate-based root canal sealer in powder/water-based gel liquid component, which is claimed to show improved performance.

**Materials and Methods:**

Thirty extracted teeth were assigned for the study. After chemomechanical preparation, the samples were separated into group I that used (TotalFill BC), and group II that utilized (MTApex) for obturation with F3 gutta-percha matching cone. Moreover, three sections of 2mm thickness were provided from each sample; and each section was assessed by Scanning Electron Microscope and Universal Testing Machine for sealer adaptation and push-out bond strength assessment, respectively. Inter-group statistically significant difference was detected via using Mann-Whitney U test, while intra-group statistical analysis was performed using Kruskal-Wallis test. Then, pairwise comparisons were done using Mann-Whitney U test with Bonferroni correction, which detected significant differences (P≤0.05).

**Results:**

Statistically, the lowest gap diameter and the highest push-out bond strength records were reported by TotalFill root canal sealer, compared to MTApex, concerning the three root sections. The coronal root section revealed lower gap diameters and higher push-out bond strength record; this is in comparison to the other sections (P≤0.05).

**Conclusions:**

MTApex demonstrated adaptation and bond strength lower than that of TotalFill. Nevertheless, inconsistent adaptation and bond strength were reported regarding the different assessed sections, where a high level of adaptation and bond strength was detected in the coronal section, middle section, and apical section, respectively.

## Introduction

Shielding healthy periapical tissue and preventing/treating pulp diseases are the main objectives of endodontic therapies [1,2]. The capability of pathogenic microorganisms to invade and persist within dentinal tubules has been documented, despite advances in root canal disinfection materials and techniques [3]. Moreover, the proven inefficiency of available instruments to access the entire maze-like root canal system is considered a significant challenge [4,5]. Due to its impact on long-term treatment outcomes, providing a leakage-proof root canal system seal stands as a primary focus of research [6,7]. In pursuit of a three-dimensional seal objective, obturation techniques and materials (solid core and sealer) have been extensively developed and investigated, parallel for optimizing the treatment outcomes [8]. However, obturation voids are still being reported; and they may compromise treatment outcomes, regardless of the techniques or materials employed [9,10]. This could be controlled by introducing thermo-plasticized gutta-percha obturation techniques, which, in turn, maximize the gutta-percha volume, and result in improving the long-term seal of root canals [11]. A short time ago, the single cone (hydraulic condensation) technique attained acceptance among dental practitioners, utilizing a greater taper gutta-percha master cone with a higher sealer volume. Claims about this technique highlight its proven simplicity, less need for specialized equipment, and quick learning curve, suggesting it could replace other procedure-sensitive methods [12]. Conversely, studies have indicated a persistent decrease in seal integrity, attributed to reduced sealer volume resulting from setting shrinkage and/or dissolution over time [13]. Calcium silicate-based sealers (CBRCSs) have been adopted since 1990; and root canal treatment sustained success have placed an exceptional emphasis on their enhanced chemical, physical, and biological properties [14,15]. Moreover, research has proven the hydraulic capacity of CBRCSs with calcium silicate and/or calcium phosphate components to interact with surrounding tissues, release calcium ions in the formation of hydroxyapatite, and infiltrate into dentinal tubules [16]. TotalFill® is a current ready-to-use CBRCS which utilizes existed dentinal tubules humidity to launch the setting reaction [17]. Along with reported cyto-compatibility, TotalFill BC sealer has demonstrated a minute setting expansion, and dentinal tubules penetration ability with enhanced bond strength, which makes it highly recommended for use in single-cone obturation techniques [18]. In addition, MTApex™ sealer is a novel calcium silicate paradigm powder/liquid form, where the liquid used for hydration is a water-based gel [19]. A powder/liquid formulation of calcium silicate sealers has been introduced to overcome the hydration problem encountered with the ready-to-use paste form [20]. Its physicochemical properties assessment has demonstrated a considerable flow and has reported the same setting time in moist and dry conditions, which make it a promising sealer for use [19]. On the contrary, there have been no studies conducted to explore MTApex sealing ability. Thus, the current study aims to assess MTApex and TotalFill sealer formulas sealing abilities when utilized in the single cone obturation technique through measuring their adaptation and strength of bond with root canal wall. The null hypothesis presented was that both calcium silicate sealer forms will show no difference regarding their adaptation and bond strength dentin wall.

## Materials and Methods

The study design has been introduced and approved by the Research Ethics Committee, Faculty of Dentistry, Taibah University (TUCDREC/240925). Moreover, the sample size was quantified using version 3.1.9.6 G*Power software (Heinrich-Heine-Universität, Düsseldorf, Germany), assuming an effect size of 0.92, alpha error of 0.05, and beta power of 0.90%. A total of 30 extracted human teeth were included and randomly allocated into two experimental groups (n=15 per group).

Thirty extracted maxillary central human incisors were allocated for the study for over one month; they confirmed free of root defects and radiographically verified single canal. Next, the samples were disinfected in sodium hypochlorite (NaOCl) 3% for 48 hours [21]. After that, they were manually cleaned under running water using periodontal curettes. The sample length was standardized to 15 mm, by horizontal reduction of the incisal edge. The working length was set 1 mm shorter than a K file #10 length (Micro mega, Boissy Saint-Léger, France), when a flush was noticed at the apical foramen. A K file # 15 was confirmed as the initial file to standardize root canal size. Additionally, root canals chemo-mechanical preparation was performed using rotary files ProTaper Gold up to # F3 (Dentsply, Tulsa Dental, Tulsa, OK, USA), with 2 mm of NaOCl 3% (Parcan™; Septodont, Plandent AB, Stockholm, Sweden) between successive files; and 5 ml of ethylenediaminetetraacetic acid (EDTA) (Merck, Frankfurt, Germany) was used for 1 minute before the canals were rinsed using a sterile saline. A matching-GP cones F3 (Dentsply maillefer) were tried to fit into root canals at the working length, after being dried utilizing paper points (MICRO-MEGA). Subsequently, the samples were categorized to two groups, based on the sealer used. Data of the investigated sealers are presented in Table 1. 

In group I (n=15), TotalFill BC sealer was delivered through the syringe into partially dried root canals slowly while simultaneously pulling the syringe out of the canal [22]. In group II (n=15), MTApex sealer was blended according to the manufacturer's guidelines, packed inside the clear Skini syringe, and dispensed into root canals with Navitip 29 gauge with slow withdrawal from the root canal [19]. A previously tried fit at working length F3-GP cones was covered with the sealer and inserted into root canals. The GP-cone excess was detached using a hot endodontic plugger and gently compacted below the cement-enamel junction. Then, the canals' access was cleaned and packed with Cavit-G (3M ESPE, Seefeld, Germany). Furthermore, obturation radiographic quality in proximal and labial views was checked. Next, the samples were incubated for 8 weeks at 37 °C/100% humidity to secure the sealers setting [23]. After that, the samples were embedded in resin; and three horizontal sections, each of 2 mm +/- 0.02 mm thickness, were sliced perpendicular to root's length, with an 8-inch diameter / 0.6 mm thickness water-cooled diamond disc (Isomet 4000, Buehler, USA). Sectioning started at 3, 7, and 11 mm from the anatomic apex. The 3-mm apical section from the anatomical apex was not included in the measurements due to increased probability of anatomical variations; and thickness accuracy was verified using digital caliper (Mituyoyo, Japan). After that, the sections were bathed lightly into distilled water and then desiccated before examinations [23]. Following that, the sections were sequentially labeled with randomized alphanumeric codes by an independent researcher.  Outcome assessment of sealer adaptations and push-out bond strength were performed by a blinded evaluator, thereby, minimizing potential bias.

*Sealer adaptation assessment*

Gap width diameter at sealer-dentin interface was assessed in the coronal, middle, and apical root sections at ×2000 of Scanning Electron Microscope-SEM (Quanta FEG 250, Netherland). The section was divided into four quadrants; and a maximum of three gap diameters, in each, were measured directly and recorded in micrometers (μm); and the mean for each section measurement was calculated [24]. 

*Evaluation of bond strength*

Measurement of push-out bond strength was performed by subjecting root sections utilizing a universal testing machine (Shimadzu, Japan). The root sections were fixed in a custom-made metal block horizontally with the coronal side facing upwards. Moreover, a circle-shaped hole was made at the middle of the block to provide room for the displacement of filling. Additionally, a compressive loading with 0.5 mm/min speed was put-on by virtue of computer-controlled machine, where the plunger tip is adjusted toward the filling, without resting on the dentin wall [23]. Bond-strength (δ) was calculated by software at time of failure using the following equation, where the maximal failure load in Newton (N) was converted to Mpa:

Push-out bond strength (MPa) = Maximum load (N)/ Adhesion area (mm^2^).

(Area) A=2 π r × h, where π is a constant= 3.14, r = root canal radius (sum of coronal and apical radii) of each section (r_1_+r_2_), and h = section height (2 mm+/-0.02 mm).

*Statistical analysis*

Measurements were recorded and exposed to SPSS software statistical analysis (SPSS Inc. Released 2008, SPSS statistics for Windows, Version 17.0, Chicago, IL, USA), which provided data mean and standard deviation. The results showed data non-normal distribution, according to the Kolmogorov-Smirnov test. Furthermore, inter-group statistically significant difference was determined via the Mann-Whitney U test. Besides, the statistical difference of the intra-group comparisons among the three root sections was measured using Kruskal-Wallis test. In addition, pairwise comparisons were performed using the Friedman test, followed by pairwise comparisons using the Wilcoxon signed-rank test with Bonferroni correction. Level of significance was set at 0.05 and adjusted accordingly for multiple comparisons.

## Results

*Sealer adaptation*

TotalFill BC® sealer (group I) demonstrated significantly lower total, coronal and middle sections gap widths than MTApex™ sealer (group II), regarding (Table 2 and Figure 1). In contrast, the statistical difference in gap width measurements of the apical root sections was non-significant. Concerning intra-group comparison of gap width measurements among the three root sections, statistical analysis recorded a significant difference among the three root sections in both groups I and II. The coronal root section revealed the lowest gap measurements, followed by the middle section. On the contrary, the apical root section reported the highest measurements.

*Push-out bond-strength*

TotalFill BC® sealer (group I) reported total, apical, middle, and coronal sections' bond strength values remarkably higher than those of MTApex™ sealer (group II) (Table 3). Intra-group statistical analysis of the three root sections recorded bond strength values within both groups revealing a statistically significant difference, where the highest values were recorded in the coronal section, followed by the middle section, and then the apical root section.

## Discussion

There have been continuous attempts to obtain three-dimensional seal of root canals through enhancement of root canal sealers, emphasizing their pivotal role [8]. Accordingly, improvement in material composition, form, and application method is an ongoing process. The present study has comprehensively investigated and compared the adaptation and bond strength of novel MTApex™ and TotalFill BC® calcium silicate sealers. To attain standardized methodology, maxillary central incisors were selected for this work; teeth where initial K file size # 15 was not fit were excluded from the study. Moreover, the samples had alike chemo-mechanical preparation, final apical size, irrigation regimen, and obturation technique [19,22]. Root canal sealers adaptation can be assessed using multiple methods, including electrochemical ones, dye penetration, fluid filtration, and others [25]. Apparently, SEM has provided an eminent resolution, depth of field, and accuracy, allowing a minute assessment of the sealers' adaptation to root canal walls through magnification and direct software measurement of the marginal gap diameters at the submicron level [24]. Furthermore, the quality of sealer adaptation and potential to resist displacement forces can be reflected through investigating the sealer-dentin interfacial bond strength, utilizing frequently bond-strength assessment method (push-out test), despite its limitation of use in clinical conditions [23]. In the current investigation, TotalFill BC sealer manifested lower gaps diameter and higher bond strength values at all root levels, in comparison with MTApex that denoted its superior adaptation potentials. Such finding is in agreement with Atmeh AR *et al*., Al-Hiyasat AS *et al*., and Mohammed SK *et al*. who disclosed a higher adaptation and bond strength in relation to TotalFill BC sealer; this is inferred by its premixed paste form, suitable consistency, and high flow (30.8 ± 0.32), along with its fine particles size (≤2 microns) that guarantee a precise delivery and adaptation to root canal walls [26,27,24]. Moreover, TotalFill BC sealer manifests a setting time of 4 hours, extending to 10 hours in dry dentine conditions, which offers additional time for the material to flow and adapt into the root canal wall [24]. Other studies have elucidated the role TotalFill BC sealer alkaline nature plays in improving its sealing ability, as reported through initial, 48 hours, and 168 hours pH values of 10.5, 12.25, and 9.3, respectively. This alkalinity permits the degrading (denaturing) effect on dentin collagen, thereby, enhancing infiltration and providing a higher adaptation and bond strength with root canal walls [28,29]. Concerning MTApex, which is the latest powder/liquid CBRCS, the manufacturer asserts that the newly established water-based gel liquid can overcome hydration problems reported in ready-to-use formulas of bioceramic sealers [19]. In the current study, MTApex has shown higher gaps diameter and lower bond strength values, when compared to TotalFill BC sealer. With reference to limited data about the novel MTApex sealer, the reported flow, which is lower (30.43 ± 2.26) than that of TotalFill BC, can influence its adaptation potential, and, consequently, bond strength, despite its considerable setting time (19.3 hours) in dry or moist conditions, according to Janini ACP *et al*. [19]. Furthermore, MTApex exhibited pH values lower than that of TotalFill BC (9.13, 10.96, and 11.34, initially, after 96, and 168 hours, respectively) which might limit its collagen-degrading ability and explain the recorded lower adaptation and bond strength to dentin [28,30]. Investigating adaptation and bond strength among the different sections in this study has revealed inconsistent results regarding the two assessed sealers. The coronal sections reported the lowest gap diameter values, followed by the middle sections. Conversely, the apical sections have manifested the highest gap diameter and lowest bond strength values. Low sealer adaptation at the apical root third is frequently reported and postulated to be an outcome of the complex nature of apical anatomy, concerning size, cross-section, and curvature, along with reported low density and irregular dentinal tubules, which restrain the attempts of disinfection, debridement, smear layer removal, and sealer adaptation [31]. On the other hand, the middle root sections showed moderate gap diameters; this can be attributed to cross-section morphological variations that might influence the adaptation of a round cross-section obturation core material [24]. Based on the available data, there are no studies that have evaluated the recently introduced sealing ability of MTApex sealer. In addition, powder particle size details are not supplied by the manufacturer or available in the literature to date. Thus, further research is required to be conducted to establish its properties and long-term sealing ability assessment. Further, future studies are recommended to focus on *in-vivo* and *in-vitro* comparative assessments to highlight the gap in knowledge and justify the claims about MTApex root canal sealer.

## Conclusions

Within the study limitations, the novel MTApex revealed adaptation and bond strength lower than those of TotalFill BC sealer. Furthermore, inconsistent adaptation and bond strength were reported at different assessed root sections, with a high value in the coronal sections. 

## Figures and Tables

**Table 1:** Table 1. Investigated sealers data.

**Sealer**	**Manufacturer**	**LOT**	**Composition**
MTApex™	Ultradent Product Inc., South Jordan,Utah, USA	Powder 2022011903	Powder: Tricalcium silicate, tricalcium-aluminate, and tantalum oxide
		Liquid 2022012202	Liquid: Water based gel
TotalFill BC™	FKG Dentaire, La Chaux-de-Fonds, Switzerland	23004SP	Zirconium oxide 35%-45%, dicalcium silicate 7%-15%, tricalcium silicate 20%-35%, calcium hydroxide 1%-4%, fillers

**Table 2:** Table 2. Mean and standard deviation (SD) of the gap width measurements in (μm) in different root sections of groups I and II .

	**Total**		**Coronal**		**Middle**		**Apical**		*P-value*
	Mean	SD	Mean	SD	Mean	SD	Mean	SD	
Group I	2.32	0.21	1.44 ^a^	0.43	2.00 ^b^	0.33	3.51^ c^	0.42	0.000**
Group II	3.07	0.22	2.38 ^a^	0.33	2.88 ^b^	0.21	3.96 ^c^	0.46	0.000**
*P-value*	0.000*		0.000*		0.000*		0.023 NS		

*: Indicates statistically significant difference between the variables using Mann-Whitney U test (P≤0.05). **: Indicates statistically significant difference between the variables using Friedman test (P≤0.05). NS: Indicates no statistically significant difference between the variables using Mann-Whitney U test (P>0.05). Different lowercase letters indicate statistically significant difference within the same row using Wilcoxon signed-rank test with Bonferroni correction (Bonferroni-adjusted P≤0.0167).

**Table 3:** Table 3. Mean and standard deviation (SD) of push-out bond strength values (MPa) in the different root sections of groups I and II^1^.

	**Total**		**Coronal**		**Middle**		**Apical**		*P-value*
	Mean	SD	Mean	SD	Mean	SD	Mean	SD	
Group I	7.04	0.30	9.26 ^a^	0.27	7.33 ^b^	0.55	4.54 ^c^	0.51	0.000**
Group II	5.72	0.35	7.79 ^a^	0.57	5.99 ^b^	0.47	3.36 ^c^	0.34	0.000**
*P-value*	0.002*		0.000*		0.000*		0.000*		

*: Indicates statistically significant difference between the variables using Mann-Whitney U test (P≤0.05). **: Indicates statistically significant difference between the variables using Friedman test (P≤0.05). NS: Indicates no statistically significant difference between the variables using Mann-Whitney U test (P>0.05). Different lowercase letters indicate statistically significant difference within the same row using Wilcoxon signed-rank test with Bonferroni correction (Bonferroni-adjusted P≤0.0167).

**Figure 1:**
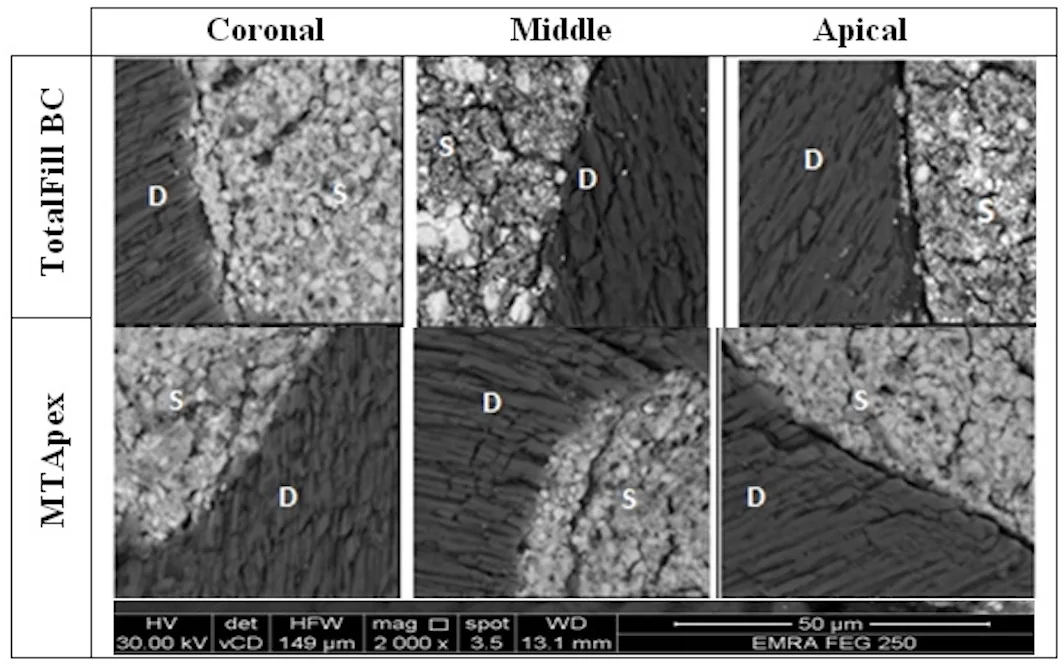
Fig. 1. SEM images at (x2000) showing the gap diameter measurements at apical, middle and coronal root sections (S: sealer& D: dentine).

## Data Availability

Declared none.
